# Phenotypic and molecular characterization of β-lactamase and plasmid-mediated quinolone resistance genes in *Klebsiella oxytoca* isolated from slaughtered pigs in Thailand

**DOI:** 10.14202/vetworld.2022.309-315

**Published:** 2022-02-13

**Authors:** Nattamol Phetburom, Parichart Boueroy, Peechanika Chopjitt, Rujirat Hatrongjit, Suphachai Nuanualsuwan, Anusak Kerdsin

**Affiliations:** 1 Department of Community Health, Faculty of Public Health, Kasetsart University, Chalermphrakiat Sakon Nakhon Province Campus, Sakon Nakhon, Thailand; 2 Department of General Sciences, Faculty of Science and Engineering, Kasetsart University, Chalermphrakiat Sakon Nakhon Province Campus, Sakon Nakhon, Thailand; 3 Department of Veterinary Public Health, Faculty of Veterinary Science, Chulalongkorn University, Bangkok, Thailand

**Keywords:** *Klebsiella oxytoca*, plasmid-mediated quinolone resistance, slaughtered pigs, Thailand, β-lactamase

## Abstract

**Background and Aim:**

Over recent years, antimicrobial-resistant *Klebsiella* species in humans, animals, food animals, food products, and agricultural environments have been the center of attention due to its role in the evolution of antimicrobial resistance. The emergence of resistance to fluoroquinolones and cephalosporins of third and higher generations in *Klebsiella oxytoca* has not received much attention in animal husbandry compared to that in *Klebsiella pneumoniae*. Reports on K. *oxytoca* are limited in the study area. Therefore, we investigated the antimicrobial susceptibility and resistance genes in K. *oxytoca* isolated from slaughtered pigs in Thailand.

**Materials and Methods:**

Microbiological examination was conducted on 384 *Klebsiella* spp. isolates recovered from slaughtered pigs in ten provinces of Thailand. Seventy-two *K. oxytoca* isolates (18.75%) were examined for antimicrobial-resistant genes (β-lactamase [*bla*_TEM_, *bla*_CTX-M_, and *bla*_SHV_]) and fluoroquinolone-resistant genes (*qnrA*, *qnrB*, *qnrC*, *qnrD*, *qnrS*, *oqxAB*, *aac(6’)-Ib-cr*, and *qepA*).

**Results:**

The most common genotype was *bla*_CTX-M_ (58/72, 80.55%), followed by *bla*_TEM_ with *bla*_CTX-M_ (7/72, 9.72%) and *bla*_TEM_ (6/72, 8.33%). The most common *bla*_CTX-M_ group was *bla*_CTX-M-1_ (19/58, 32.76%), followed by *bla*_CTX-M-9_ (1/58, 1.72%). Plasmid-mediated quinolone resistance genes were identified in 13 (18.05%) isolates: *qnrS* (16.70%) and *qnrB* (1.4%). All 13 isolates had *qnrS* transferable to an *Escherichia coli* recipient, whereas *qnrB* was not detected in any transconjugants. Either *bla*_CTX-M_ or *bla*_TEM_ harbored by one *K. oxytoca* strain was transferable to an *E. coli* recipient. Analysis of antimicrobial susceptibility revealed that more than 90% of the *bla*_CTX-M_-carrying *K. oxytoca* isolates were susceptible to chloramphenicol, trimethoprim, ceftazidime, cefepime, cefotaxime, amoxicillin-clavulanic acid, piperacillin–tazobactam, and fosfomycin. All *K. oxytoca* isolates (13) harboring *qnr* were susceptible to carbapenem and ceftriaxone; however, 43 (74.13%) of the *K. oxytoca* isolates harboring *bla*_CTX-M_ exhibited extended-spectrum β-lactamase activity. Most of the *K. oxytoca* isolates from pigs were highly resistant to ampicillin, azithromycin, and gentamicin.

**Conclusion:**

To prevent further transmission of *Klebsiella* spp. Between food animals and humans, strict control of antibiotic use in clinical and livestock settings is necessary along with routine disinfection of the livestock environment and efforts to increase awareness of antimicrobial resistance transmission.

## Introduction

Extended-spectrum β-lactamase (ESBL) was first isolated from *Klebsiella* species and later from *Escherichia coli*, *Pseudomonas aeruginosa*, *Serratia marcescens*, and other Gram-negative bacilli [1,2]. ESBL can confer resistance to a variety of b-lactam antibiotics, including penicillins and broad-spectrum cephalosporins with an oxyimino side chain, such as cefotaxime, ceftriaxone, and ceftazidime, but not often to carbapenems or cephamycins, such as cefoxitin [3]. ESBL-producing Gram-negative bacilli have rapidly spread and become a serious threat to human health worldwide, resulting in significant morbidity and mortality [4]. Various types of β-lactamase genes (including ESBL genes), such as *bla*_CTX-M_, *bla*_SHV_, and *bla*_TEM_, have been reported in *K. oxytoca* strains obtained from patients and healthy persons [5]. Within the *bla*_CTX-M_ family, *bla*_CTX-M-2_-carrying *K. oxytoca* strains have been identified in milk samples obtained from cows with mastitis in Japan [6]. In a study conducted in Italy, *bla*_CTX-M-9_, *bla*_SHV-12_, and *bla*_DHA-1_ genes were detected in *K. oxytoca* isolates [7].

Plasmid-mediated resistance to quinolones (PMQR) genes, including *qnrA*, *qnrB*, *qnrS*, *qnrC*, and *qnrD* genes, has been demonstrated to play a significant role in quinolone resistance by protecting DNA gyrase and topoisomerase IV from the inhibitory activity of quinolones [8,9]. Aminoglycoside acetyltransferase (*aac(6’)-Ib-cr*) gene has been shown to enzymatically modify fluoroquinolones [9]. The production of efflux pumps is enhanced by *qepA*, *acrAB*, and *oqxAB* genes, which also contribute to quinolone and fluoroquinolone resistance [10]. *Klebsiella oxytoca* isolated from cats and dogs in Italy were positive for *qnr* genes (*qnrA1* and *qnrB4*), and one *K. oxytoca* isolate was positive for the *aac(6’)-Ib-cr* gene [7].

The spread of *K. ocytoca* strains carrying β-lactamase and PMQR genes may be a threat to the “One Health” program. In Thailand, where antibiotics are used without restriction in the healthcare, livestock, and food production sectors, research on the epidemiology of antibiotic-resistant bacteria in food animals and its associated public health implications has been conducted in common bacteria, such as *E. coli*, *Klebsiella pneumoniae*, and *Salmonella* spp.

However, reports on *K. oxytoc*a are limited in the study area. Therefore, we investigated the antimicrobial susceptibility and resistance genes in *K. oxytoca* isolated from slaughtered pigs in Thailand.

## Materials and Methods

### Ethical approval

Ethical review and approval were not required for this study as the samples were collected from slaughtered pigs at the slaughterhouses as per standard collection procedure.

### Study period and location

**Figure-1:**
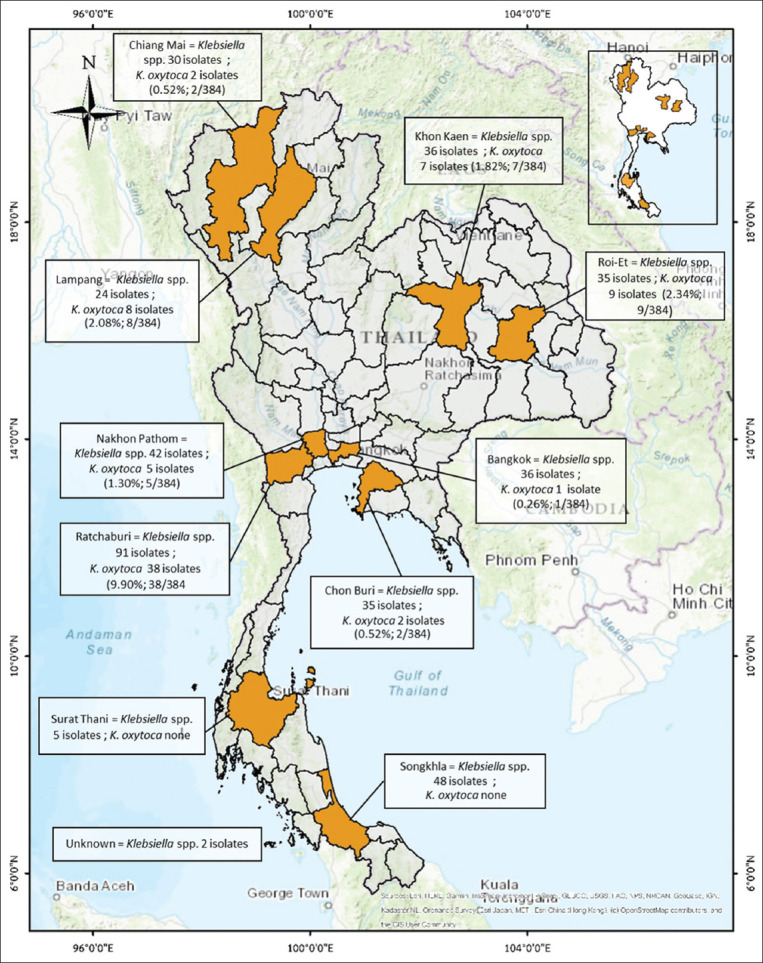
Locations for samples collection from slaughterhouses in 10 provinces nationwide: Bangkok, Nakhon Pathom, Lop Buri, Chiang Mai, Lampang, Chon Buri, Roi-Et, Khon Kaen, Surat Thani, and Songkhla [Source: A geographical information system (GIS) software QGIS (version 2.18.28) was used to create a study map].

The study was conducted from October 2014 to September 2015. The sampling locations were the slaughterhouses in 10 provinces nationwide: Bangkok, Nakhon Pathom, Lop Buri, Chiang Mai, Lampang, Chon Buri, Roi-Et, and Khon Kaen. Surat Thani, and Songkhla (Figure-1).

### Bacterial isolates and identification

We followed the procedures for isolation and identification of *Klebsiella* spp. as described by Phetburom *et al*. [11]. We recovered 384 isolates of *Klebsiella* spp. from slaughtered pigs. Four slaughterhouses were randomly selected from each province, and 50 carcass surface swab samples were randomly collected from each slaughterhouse, which results in a sample size of 2000 swab samples. Using a single swab for each carcass, 400 cm^2^ of one side of the carcass was swabbed. The swab samples were immediately stored on ice in zip-lock bags and transported to the microbiological laboratory. Isolation and identification of *Klebsiella* spp. were performed using 10-fold serial dilutions of swab samples achieved using buffered peptone water (BPW; pH 7.2). Each diluted BPW sample was spread onto MacConkey agar (Difco Laboratories, Detroit, Mich. USA) and incubated at 37°C for 24 h. The presumptive identification of *Klebsiella* species was confirmed using conventional biochemical tests, including motility, Voges-Proskauer, ornithine decarboxylation, lysine decarboxylation, malonate, and O-nitrophenyl-β-D-galactopyranoside [12]. In total, 384 Klebsiella isolates were recovered and all isolates were stored at −80°C in a laboratory freezer until use in this study. The presumptive *Klebsiella* species for up to five colonies were cultured on MacConkey agar (Difco Laboratories) and their DNA was prepared using ZymoBIOMICS™ DNA Miniprep Kits (Zymo Research Corp., CA, USA) according to the manufacturer’s protocols. Polymerase chain reaction (PCR) was performed using a thermal cycler to identify *K. oxytoca*, as described elsewhere [13]. The PCR program included initial denaturation at 95°C for 3 min, 30 cycles of denaturation at 95°C for 30 s, annealing at 55°C for 45 s, extension at 72°C for 1 min, and a final cycle of amplification at 72°C for 7 min.

### Detection of β-lactamase genes

Isolates carrying β-lactamase genes (*bla*_TEM_, *bla*_CTX-M_, and *bla*_SHV_) were detected through multiplex PCR using a thermal cycler with a PCR program that included initial denaturation at 95°C for 3 min, 30 cycles of denaturation at 95°C for 30 s, annealing at 55°C for 30 s, extension at 72°C for 1 min, and amplification at 72°C for 7 min [14]. The *bla*_CTX-M_-harboring *K. oxytoca* was classified into CTX-M groups (*bla*_CTX-M-1_, *bla*_CTX-M-2_, *bla*_CTX-M-5_, *bla*_CTX-M-8_, *bla*_CTX-M-9_, and *bla*_CTX-M-25_ groups) using multiplex PCR [15]. PCR amplification was performed using a thermal cycler with a PCR program of denaturation at 94°C for 5 min, 30 cycles of denaturation at 94°C for 25 s, annealing at 52°C for 40 s, extension at 72°C for 50 s, and amplification at 72°C for 6 min.

### Detection of plasmid-mediated quinolone resistance genes

PMQR genes (*qnrA*, *qnrB*, *qnrC*, *qnrD*, *qnrS*, *oqxAB*, *aac*(6’)*-Ib-cr*, and *qepA)* were determined using multiplex PCR [16]. PCR amplification was performed using a thermal cycler with a PCR program of denaturation at 95°C for 3 min, 30 cycles of denaturation at 95°C for 30 s, annealing at 63°C for 90 s, extension at 72°C for 90 s, and amplification at 72°C for 10 min.

### Antimicrobial susceptibility testing

*K. oxytoca* isolates harboring *bla*_CTX-M_, *qnrS*, or *qnrB* were further investigated for antimicrobial susceptibility using the disk diffusion method according to the 2021 Clinical and Laboratory Standards Institute guidelines [17]. The antimicrobial disks used in the assay were loaded with ampicillin (10 μg), gentamicin (10 μg), amikacin (30 μg), amoxicillin–clavulanic acid (30 μg), piperacillin-tazobactam (110 μg), ceftriaxone (30 μg), cefepime (30 μg), cefotaxime (30 μg), ceftazidime (30 μg), ertapenem (10 μg), imipenem (10 μg), meropenem (10 μg), ciprofloxacin (5 μg), levofloxacin (5 μg), chloramphenicol (30 μg), tetracycline (30 μg), fosfomycin (200 μg), nitrofurantoin (300 μg), azithromycin (5 μg), or trimethoprim (5 μg). *E. coli* ATCC 25922 was used as the control.

### Detection of ESBL production based on combination disk test

The production of ESBL was tested for in 58 *bla*_CTX-M_-harboring *K. oxytoca* isolates using the combined disk method and separate commercial disks containing cefotaxime (30 μg) and ceftazidime (30 μg) with or without clavulanic acid (10 μg) [17]. An increase in zone size ≥5 mm for cefotaxime and ceftazidime with or without clavulanic acid was considered to indicate ESBL production [17].

### Detection of ESBL Nordmann–Dortet–Poirel (NDP) assay

The *bla*_CTX-M_-harboring *K. oxytoca* isolates were tested for extended-spectrum β-lactamase activity using the ESBL NDP assay [18]. One calibrated inoculated loop (10 μL) of the tested strain was briefly suspended in 100-μL B-PER II^®^ Bacterial Protein Extraction Reagent (Thermo Scientific, USA) buffer and centrifuged at room temperature for 5 min. The supernatant (30 μm) was mixed with 100 μL of a 1-mL solution of pH 7.8 phenol red solution with or without 6 mg of purified cefotaxime sodium salt (Tokyo Chemical Industry Co., Ltd, Japan), incubated at 37°C for 2 h, and observed for color change. Extended-spectrum β-lactamase-producing strains were identified as they broke down cefotaxime into acidic products, changing the color of the phenol red indicator to yellow.

### Conjugation assay

Conjugation assays were performed using all isolates of *K. oxytoca* harboring *bla*_CTX-M_ and *qnrS* as donors and streptomycin-resistant *E. coli* UB1637 as recipients, as described elsewhere [19]. Mueller–Hinton agar plates supplemented with 3200 mg/mL of streptomycin were used to select transconjugants. Transconjugants were confirmed as *E. coli* using PCR [20], and PCR was used to determine the presence of ESBL and PMQR genes.

## Results

### Detection of β-lactamase gene in *K. oxytoca*

Of the 384 *Klebsiella* spp., 72 were identified as *K. oxytoca* (18.75%) using PCR (Figure-1). In all *K. oxytoca* isolates, *bla*_TEM_, *bla*_CTX-M_, and *bla*_SHV_-β-lactamase genes were detected. We detected 64 isolates (88.89%) carrying β-lactamase genes. Among these, the majority only carried *bla*_CTX-M_ (51/72, 70.83%), and seven carried *bla*_TEM_ and *bla*_CTX-M_ genes (7/72, 9.72%), as presented in Table-1. Of the 58 isolates of *bla*_CTX-M_-harboring *K. oxytoca* (51 carrying only *bla*_CTX-M_ and 7 carrying *bla*_CTX-M_ and another resistance gene), 32.76% carried *bla*_CTX-M-1_ (19/58), 1.72% carried *bla*_CTX-M-9_ (1/58), and 65.52% carried *bla*_CTX-M_ of an unknown group (38/58), as presented in Table-1.

**Table 1:** Characterization of antimicrobial resistance genes in 72 *Klebsiella oxytoca* isolated from slaughtered pigs in Thailand.

Species	β-lactamase genes (%)					PMQR (%)	

	β-lactamase genes (%)			CTX-M group (%)			
	
	*bla_TEM_*	*bla_CTX-M_*	*bla_TEM_+bla_CTX-M_*	Group 1	Group 9	*qnr S*	*qnr B*
*Klebsiella oxytoca*	6 (8.33)	51 (70.83)	7 (9.72)	19 (32.76)	1 (1.72)	12 (16.67)	1 (1.38)

PMQR=Plasmid-mediated quinolone resistance

### Detection of plasmid-mediated quinolone resistance genes

Among the PMQR genes detected, *qnrS* was found in 16.67% (12/72) of *K. oxytoca* isolates (Table-1). One isolate had *qnrB* (1.4%). Of the 12 *qnrS*-carrying isolates, 8 had only *qnrS*, 2 had *qnrS* and *bla*_CTX_ of an unknown group, and 2 had *qnrS* and *bla*_TEM_ (Table-1).

### Transferability of β-lactamase and PMQR genes

The 61 *K. oxytoca* isolates carrying *bla*_CTX-M_, *qnr*, or both were subjected to conjugation assay using streptomycin-resistant *E. coli* UB1637 as the recipient. Not all *K. oxytoca* donors carrying only *bla*_CTX-M_ (n=50) transferred resistance to recipient *E. coli* cells. On the contrary, *K. oxytoca* isolates carrying only *qnrS* (n=8) and those harboring either *qnrS* with *bla*_CTX-M_ or *qnrS* with *bla*_TEM_ (n=4) successfully transferred resistance to recipient *E. coli* (Table-2). Among these transconjugants, *qnrS*, *qnrS* with *bla*_CTX-M_, and *qnrS* with *bla*_TEM_ were successfully transferred, whereas *qnrB* was not detected in any of the transconjugants in this study (Table-2).

**Table 2:** Characteristics and antimicrobial resistance profiles of donor and transconjugant of 13 *Klebsiella oxytoca* harboring PMQR genes.

ID	Pattern of PMQR donor	Gene transfer	

		β-lactamases	PMQR
19 RB 111.1	*qnrS*	-	*qnrS*
26 RB 116.2	*bla*_TEM_+*bla*_CTX-M_+*qnrS*	-	*qnrS*
1 KK 1.2	*bla*_TEM_+*qnrS*	*bla*_TEM_	*qnrS*
60 KK 111.3	*bla*_TEM_+*qnrS*	*bla*_TEM_	*qnrS*
33 LP 36.3	*bla*_CTX-M_+*qnrS*	-	*qnrS*
41 LP 45.3	*bla*_CTX-M_+*qnrS*	-	*qnrS*
44 LP 53.2	*bla*_CTX-M_+*qnrS*	-	*qnrS*
47 LP 51.2	*bla*_CTX-M_+*qnrS*	*bla*_CTX-M_	*qnrS*
50 LP 54.2	*bla*_CTX-M_+*qnrS*	-	*qnrS*
51 LP 55.2	*bla*_CTX-M_+*qnrB*	-	*-*
54 LP 61.1	*bla*_CTX-M_+*qnrS*	*bla*_CTX-M_	*qnrS*
61 LP 54.2	*bla*_CTX-M_+*qnrS*	-	*qnrS*
35 CM 72.2	*bla*_CTX-M_+*qnrS*	-	*qnrS*
Total		4 (30.77%)	12 (92.31%)

PMQR=Plasmid-mediated quinolone resistance

### Antimicrobial susceptibility testing

**Figure-2:**
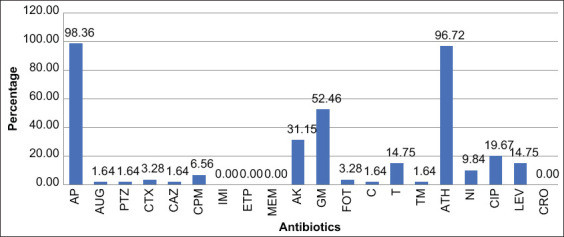
Antimicrobial resistance of 61 *bla*_CTX-M_*-*harboring or *qnrS*-harboring *Klebsiella oxytoca* isolated from slaughtered pigs. Antimicrobial resistance of 61 blaCTX-M-harboring or qnrS-harboring Klebsiella oxytoca isolated from slaughtered pigs. AP=Ampicillin, GM=Gentamicin, AK=Amikacin, AUG=Amoxicillin/clavulanic acid, PTZ=Piperacillin-tazobactam, CPM=Cefepime, CTX=Cefotaxime, CIP=Ciprofloxacin, LEV=Levofloxacin, ETP=Ertapenem, IMI=Imipenem, MEM=Meropenem, CRO=Ceftriaxone, CAZ=Ceftazidime, C=Chloramphenicol, T=Tetracycline, FOT=Fosfomycin, NI=Nitrofurantoin, ATH=Azithromycin, TM=Trimethoprim.

A total of 20 antimicrobial agents were tested on 61 *K. oxytoca* isolates harboring *bla*_CTX-M_ and/or *qnrS* genes. All isolates were susceptible to carbapenems (ertapenem, imipenem, and meropenem) and ceftriaxone (Figure-2 and Table-3). More than 90% of *K. oxytoca* isolates were susceptible to chloramphenicol, trimethoprim, ceftazidime, cefepime, cefotaxime, amoxicillin–clavulanic acid, piperacillin–tazobactam, and fosfomycin. Almost all *K. oxytoca* isolates were highly resistant to ampicillin (98.36%) and azithromycin (96.72%). Resistance was also recorded to gentamicin (52.46%), amikacin (31.15%), ciprofloxacin (19.67%), tetracycline (14.75%), nitrofurantoin (9.84%), and levofloxacin (14.75%), as presented in Figure-2 and Table-3. An ESBL-producing phenotype based on the combination disk test showed that 100% of *bla*_CTX-M-_carrying *K. oxytoca* were non-ESBL producers. The ESBL NDP assays revealed that 74.13% (43/58) of the *K. oxytoca* isolates harboring *bla*_CTX-M_ exhibited β-lactamase activity. Of the 13 *qnrS*- and *qnrB*-harboring *K. oxytoca* isolates, 9 (69.23%) showed resistance to fluoroquinolone: About 66.67% for ciprofloxacin 88.88% for levofloxacin and 55.56% for both.

**Table 3:** Characteristics and antimicrobial resistance profiles of *Klebsiella oxytoca* isolated from slaughtered pigs in Thailand.

Pattern of resistance genes	Total (%)	Pattern of antimicrobial resistance	Total (%)
*bla*_CTX-M_	42 (68.85)	AP-ATH	10 (16.39)
		AP-ATH-GM	8 (13.11)
		AP-ATH-GM-AK	6 (9.84)
		AP-ATH-GM-AK-NI	3 (4.92)
		AP-ATH-CIP	2 (3.28)
		AP-ATH-AUG-NI-CIP-TM	1 (1.64)
		AP-ATH-GM-CTX-CPM	1 (1.64)
		AP-ATH-GM-NI-CIP	1 (1.64)
		AP-ATH-GM-NI	1 (1.64)
		AP-ATH-CTX-AK	1 (1.64)
		AP-ATH-T-CIP	1 (1.64)
		AP-ATH-AK	1 (1.64)
		AP-ATH-FOT	1 (1.64)
		AP-ATH-C	1 (1.64)
		AP-ATH-LEV	1 (1.64)
		AP-FOT	1 (1.64)
		AP	1 (1.64)
		ATH	1 (1.64)
*bla*_CTX-M_ *+qnr S*	8 (13.11)	AP-ATH-GM-T	2 (3.28)
		AP-ATH-GM-CPM-T-CIP-LEV	2 (3.28)
		AP-ATH-GM-AK-CAZ-T-CIP	1 (1.64)
		AP-ATH-CPM-T-CIP-LEV	1 (1.64)
		AP-ATH-GM-T-CIP-LEV	1 (1.64)
		AP-ATH-GM-AK	1 (1.64)
*bla*_TEM_ *+bla*_CTX-M_	6 (9.84)	AP-ATH	4 (6.55)
		AP-ATH-GM-AK	1 (1.64)
		AP-ATH-GM-AK-PTZ	1 (1.64)
*bla*_TEM_ *+bla*_CTX-M_ *+qnr S*	1 (1.64)	AP-ATH-GM-AK	1 (1.64)
*bla*_CTX-M_ *+qnr B*	1 (1.64)	AP-ATH-GM-AK-T-LEV	1 (1.64)
*bla*_TEM_ *+qnr S*	2 (3.28)	AP-ATH-LEV	2 (3.28)
*qnr S*	1 (1.64)	AP-ATH-AK-CIP-LEV	1 (1.64)

AP=Ampicillin, ATH=Azithromycin, GM=Gentamicin, AK=Amikacin, NI=Nitrofurantoin, CIP=Ciprofloxacin, AUG=Amoxicillin/clavulanic acid, TM=Trimethoprim, CPM=Cefepime, FOT=Fosfomycin, C=Chloramphenicol, LEV=Levofloxacin, T=Tetracycline, CAZ=Ceftazidime, PTZ=Piperacillin-tazobactam

## Discussion

Our study revealed that the majority of β-lactamase-carrying *K. oxytoca* isolates possessed *bla*_CTX-M_, especially *bla*_CTX-M-1_. In the current study, we did not detect *bla*_SHV_. The dissemination of *bla*_CTX-M-2_ among *K. oxytoca* isolates collected from pigs has been reported [21,22], and *bla*_CTX-M-15_ and *bla*_TEM-1_ were detected in *K. oxytoca* isolates collected from six provinces in China [23]. *K. oxytoca* isolates harboring *bla*_CTX-M_ were highly susceptible to cefotaxime, ceftazidime, and cefepime (93.1-98.3%) and 100% susceptible to ceftriaxone. Combination disk assay revealed that they were non-ESBL producers; however, ESBL NDP assay demonstrated that 43 (74.1%) of these isolates exhibited β-lactamase activity on cefotaxime. Another study observed that *K. oxytoca* showed ostensibly positive resistance to cefotaxime and cefepime and rare resistance to ceftazidime, with only borderline resistance to these cephalosporins (MIC 2–8 mg/L), suggesting that there was hyperproduction of K1 (KOXY) chromosomal β-lactamase rather than ESBL production [24]. The positive result for ESBL NDP assay but the negative result for combination disk assay seen in our study may suggest the presence of K1 β-lactamase in this organism.

The current study indicated that *K. oxytoca* isolated from slaughtered pigs was highly susceptible to cefoxitin (96.72%), ceftazidime (98.36%), and cefepime (93.44%). Hossain *et al*. [25] reported that *K. oxytoca* strains isolated from pet turtles in Korea were highly susceptible to cefoxitin (81.25%), ceftazidime (80.00%), and cefepime (80.77%), and based on antimicrobial susceptibility testing results, 98.3% of the *K. oxytoca* strains were resistant to ampicillin. *Klebsiella* spp., especially *K. pneumoniae* and *K. oxytoca*, produce different chromosomal β-lactamases, leading to natural resistance to penicillins [26].

In the current study, *qnrS* and *qnrB-*harboring *K. oxytoca* strains were widely distributed in the isolates and were resistant to ciprofloxacin (66.67%), levofloxacin (88.9), or both (55.56%). These results contrast with the findings of Hossain *et al*. [25] that *K. oxytoca* strains harboring *qnrA*, *qnrA* with *qnrB*, and *qnrB* with *qnrS* were highly susceptible to ciprofloxacin (100%) and ofloxacin (100%). In addition, *qnrB* and *qnrA* were the most prevalent genes (37.50%) in *K. oxytoca* strains isolated from pet turtles in Korea [26]. In this study, all isolates demonstrated capacity to transfer *qnrS* to an *E. coli* recipient. Literak *et al*. [27] found that *qnrS* genes were carried by plasmids of the N, X1, and X2 incompatibility groups and were transferable by conjugation to *E. coli* and/or *Salmonella* spp. This could support that *qnrS-*carrying *K. oxytoca* in the current study may have carried the *qnrS* genes on plasmids.

This study found that some *qnrS*, *qnrS* with *bla*_CTX-M_, and *qnrS* with *bla*_TEM_ genes were transferred to *E. coli* recipients. However, most of the *bla*_CTX-M_ in *K. oxytoca* donors were not transferred to recipient *E. coli* cells. On the contrary, Zhang *et al*. [23] reported that the *bla*_CTX-M_ and *bla*_TEM_ genes were successfully transferred from *K. oxytoca* to *E. coli* 600. This difference may be because *bla*_CTX-M_ genes may be located on chromosomes or nonconjugative plasmids. Thus, the characterizations to understand the origin, evolution, and spread of antimicrobial resistance genes are required for further investigation using whole-genome analysis.

## Conclusion

*bla*_CTX-M-1_ and *qnrS* were the predominant resistance genes in *K. oxytoca* strains isolated from slaughtered pigs in Thailand. Further study and ongoing efforts are necessary to develop a complete understanding of the epidemiology, risk factors, transmission dynamics, and public health implications associated with food animals as reservoirs of antimicrobial-resistant bacteria.

## Authors’ Contributions

PB and AK: Contributed to conceptualization and design of the study. NP: Performed the laboratory work. PB, PC, and RH: Performed validation. SN: Analyzed and interpreted the data. PB: Prepared and wrote the original draft. PB and AK: Reviewed and edited the manuscript. All authors read and approved the final manuscript.
